# Long non-coding RNA (lncRNA) nuclear enriched abundant transcript 1 (NEAT1) regulates fibroblast growth factor receptor substrate 2 (FRS2) by targeting microRNA (miR)-29-3p in hypertrophic scar fibroblasts

**DOI:** 10.1080/21655979.2021.1959221

**Published:** 2021-08-20

**Authors:** Qinghua Wu, Junjie Chen, Ziming Tan, Dehuai Wang, Jianwen Zhou, Dan Li, Ying Cen

**Affiliations:** a The Department of Plastic and Burn Surgery of West China Hospital, Sichuan University, Chengdu, China; b Burn and Plastic Surgery, Chengdu Second People's Hospital, Chengdu, Sichuan

**Keywords:** Hypertrophic scar fibroblast, lncRNA NEAT1, miR-29-3p, inhibitor of growth family member 2

## Abstract

Long non-coding RNAs (lncRNAs) play crucial roles in human diseases. However, the detailed role of lncRNAs in hypertrophic scar fibroblasts (HSFs) is inadequately understood. This study aimed to investigate the potential role of lncRNA nuclear enriched abundant transcript 1 (NEAT1) in hypertrophic scarring. Expression of lncRNAs, miRNAs, and genes were detected by polymerase chain reaction; protein expression was evaluated using western blotting. Cellular function was determined using the CCK-8 assay. The interaction between microRNA (miR)-29-3p and NEAT1 or fibroblast growth factor receptor substrate 2 (FRS2) was verified by luciferase and RNA pull-down assays. The results showed that NEAT1 was overexpressed in the hypertrophic dermis and in HSFs. However, knockdown of NEAT1 suppressed the proliferation and extracellular matrix (ECM) production of HSFs. Moreover, NEAT1 functioned as a competing endogenous RNA to upregulate FRS2 by sponging miR-29-3p. Downregulation of miR-29-3p or overexpression of FRS2 antagonized the effects of NEAT1 knockdown and promoted HSF proliferation and ECM release. In conclusion, NEAT1 knockdown protected against hypertrophic scarring by modulating the miR-29-3p/FRS2 axis, which is a viable target in scar treatment.

## Introduction

Hypertrophic scarring is a fibrotic disorder that results from severe skin trauma or burn injuries [1]. Hypertrophic scars are usually accompanied by pruritus, pain, and contractures, which negatively affect patients’ quality of life. Recently, different methods have been used to prevent or treat hypertrophic scars, such as pressure therapy, radiation therapy, surgical excision, and steroid injections. However, these methods remain sub-optimal [2]. Therefore, the mechanism underlying hypertrophic scar formation needs to be fully understood, so that proper treatments can be developed.

Emerging evidence has indicated that hypertrophic scars might arise from activated fibroblasts with exceeding capability for collagen synthesis and cell proliferation [3,4]. Hypertrophic scars are associated with the abundant expression of α-SMA-producing myofibroblasts coupled with collagen-III and collagen-I [5].

Long non-coding RNAs (lncRNAs) are a category of transcripts with over 200 nucleotides that have no protein-coding potential [6]. Numerous studies have shown the important role played by lncRNAs in various diseases, including fibrotic disorders [7,8]. The lncRNA NEAT1 is involved in human cancers [9,10] and has been reported to facilitate the migration, invasion, and proliferation of melanoma cells by regulating miR-495-3p and E2F3 [11]. Moreover, NEAT1 knockdown promotes the synthesis of collagen-II in human nucleus pulposus cells and collagen fiber hyperplasia in AHS mice [12,13]. Downregulation of NEAT1 suppresses keloid fibroblast progression [14]. However, the potential role of NEAT1 in hypertrophic scarring has not been fully elucidated.

MicroRNAs (miRNAs) are small non-coding RNAs [15]. miRNAs modulate various biological processes, such as cell invasion, proliferation, stress response, and differentiation [16]. Previous reports have shown that the aberrant expression of miRNAs, such as miR-181a [17], miR-203 [18], and miR-141-3p [19], is involved in keloid development, an essential component of hypertrophic scar formation. Additionally, miR-29 inhibits the growth of hypertrophic scar fibroblasts (HSF) [20].

Fibroblast growth factor receptor substrate 2 (FRS2) is involved in various cellular processes and is critical for morphogenesis and hypertrophic growth during heart development [21]. FRS2 is involved in collagen II and collagen X expression in chondrocytes [22]. However, the knockdown of FRS2 suppresses melanogenesis by inhibiting the paracrine effects of keratinocytes and fibroblasts [23]. Nonetheless, the potential role of FRS2 in hypertrophic scarring remains unclear.

Although NEAT1 has a direct regulatory function over miR-29-3p and FRS2, the mechanism underlying their function in HSFs remains unclear. In this study, we identified the role played by NEAT1 in HSFs via the miR-29-3p/FRS2 axis.

## Materials and methods

### Clinical samples

Hypertrophic scar tissues and adjacent normal tissues were collected from patients with hypertrophic scarring at the West China Hospital, Sichuan University in May 2019–April 2020. Tissues were immediately stored in liquid nitrogen. This study was approved by the West China Hospital of Sichuan University. All patients provided informed consent.

### Cell culture

HSFs were isolated from the clinical samples and were incubated in DMEM containing 10% FBS and 1% penicillin/streptomycin (Gibco, Califonia, USA) under 5% CO_2_ at 37°C [24].

### Cell transfection

si-NEAT1 and its control (si-NC), miR-29-3p mimic (miR-29-3p), and mimic nc; NEAT1 overexpression plasmids (NEAT1); and the empty vector (Vector) were obtained from GenePharm (Shanghai, China). Cells were transfected using Lipofectamine 2000 (Invitrogen, Califonia, USA) in accordance with the manufacturer’s instructions when cell confluence reached to over 60% [25].

### Cell Counting Kit-8 (CCK-8) assay

After transfection, the cells were plated into 96-well plates and cultured for 24 h [24]. Thereafter, CCK-8 reagents (20 µL/well) were added, and the cells were cultured for another 4 h. The absorbance values at 450 nm were detected using a SpectraMax M5 microplate reader.

### Quantitative real-time polymerase chain reaction (qRT-PCR)

Total RNA was extracted using TRIzol reagent. RNA was reverse transcribed into cDNA according to the kit instructions [26]. PCR was performed using SYBR Green Taq II (Bio-Rad, USA). The results were determined using the 2^−∆∆Ct^ method [23]. Glyceraldehyde 3-phosphate dehydrogenase (GAPDH) and U6 were used as internal references for mRNA and miRNA, respectively. Each independent experiment was performed in triplicate. Primer sequences used were as followed: NEAT1 (forward: 5ʹ-GGATATGCCCTTCATCCTGTGACGC-3ʹ; reverse: 5ʹ-TCCACACGTCCACTGTCCCAAAATC-3ʹ), α-SMA (forward: 5ʹ-GACCGAATGCAGAAGGAGAT-3ʹ; reverse: 5ʹ-CCACCGATCCAGACAGAGTA-3ʹ), COL1A1 (forward: 5ʹ-CTGCTGGACGTCCTGGTGAA-3ʹ; reverse: 5ʹ-ACGCTGTCCAGCAATACCTTGAG-3ʹ), COL1A2 (forward: 5ʹ-GAGGGCAACAGCAGGTTCACTTA-3ʹ; reverse: 5ʹ-TCAGCACCACCGATGTCCAA-3ʹ), COL3A1 (forward: 5ʹ-CCCACTATTATTTTGGCACAACAG-3ʹ; reverse: 5ʹ-AACGGATCCTGAGTCACAGACA-3ʹ), ki67 (forward: 5ʹ-ACGCCTGGTTACTATCAAAAGG-3ʹ; reverse: 5ʹ-CAGACCCATTTACTTGTGTTGGA-3ʹ), PCNA (forward: 5ʹ-CCTGCTGGGATATTAGCTCCA-3ʹ; reverse: 5ʹ-CAGCGGTAGGTGTCGAAGC-3ʹ), miR-29-3p (forward: 5ʹ-ACCCCTTAGAGGATGACTGAT-3ʹ; reverse: 5ʹ-AACCGATTTCAGATGGTGCT-3ʹ), FRS2 (forward: 5ʹ-ATGGGAATGAGTTAGGTTCTGGC-3ʹ; reverse: 5ʹ-GCGGGTGTATAAAATCAGTTCTGTG-3ʹ), U6 (forward: 5ʹ-CTCGCTTCGGCAGCACA-3ʹ; reverse: 5ʹ-AACGCTTCACGAATTTGCGT-3ʹ), and GAPDH (forward: 5ʹ-GGAGCGAGATCCCTCCAAAAT-3ʹ; reverse: 5ʹ-GGCT GTTGTCATACTTCTCATGG-3ʹ).

### Western blot assay

Total protein was collected using the RIPA buffer (Beyotime, Shanghai, China). Protein concentration was measured using a bicinchoninic acid kit. The proteins (15 µg) were then isolated using 10% SDS-PAGE and transferred onto PVDF membranes (Millipore). After sealing with 5% nonfat milk, the membranes were incubated with primary antibodies and with horseradish peroxidase-conjugated secondary antibodies. Protein bands were detected using an ECL reagent (EMD Millipore) [27].

### Dual-luciferase reporter assay

The targets of NEAT1 and miR-29-3p were predicted using the online databases Starbase3.0 and TargetScan7.2 [28]. Wild-type and mutant NEAT1 or FRS2 containing the binding sites of miR-29-3p were provided by Jikai Gene, Shanghai. Cells were co-transfected with miR-29-3p mimic or mimic nc and NEAT1 3′UTR wt or mutant (FRS2 3′UTR wt or mutant) using Lipofectamine 2000 for 48 h. Luciferase activity was detected using a dual luciferase activity reporter assay kit (Promega). Renilla luciferase activity was normalized to firefly luciferase activity.

### RNA pull-down assay

The miR-29-3p pull-down assay was performed as previously described [29]. Briefly, biotinylated negative control and miR-29-3p were acquired from GenePharm (Shanghai, China). HSFs were cultured with either biotin-NC or biotin-miR-29-3p and incubated with streptavidin-coupled beads. Subsequently, the precipitated RNA was eluted. The results were analyzed by qRT-PCR.

### In vivo *assay*

Male C57BL/6 mice (6–8 weeks, 18–25 g) were provided by Southwest Medical University. The mice were housed under standard conditions. Mice were randomly divided into four groups: control group, hypertrophic scarring (HS) group, HS+sh-NC group, and HS+sh-NEAT1 group. Mice in the HS group were injected with 2 × 8 mg/mL of bleomycin sulfate using an osmotic pump to establish the HS model [30]; mice in the HS+sh-NC group with 2 × 8 mg/mL of bleomycin sulfate and 2 mg/kg of sh-NC, and mice in the HS+sh-NEAT1 group with 2 × 8 mg/mL of bleomycin sulfate and 2 mg/kg of sh-NEAT1. After 28 days, the mice were anesthetized by intraperitoneal injection of pentobarbital sodium. Hematological analysis was performed using hematoxylin and eosin staining.

### Statistical analysis

Data were evaluated using GraphPad Prism 7 and are presented as the mean ± SD. Differences between two groups were analyzed using Student’s *t*-test and ANOVA analysis was for multiple groups. Correlation was evaluated using Spearman’s analysis. Statistical significance was set at *p* < 0.05.

## Results

### NEAT1 was upregulated in HSFs

**Figure 1.**
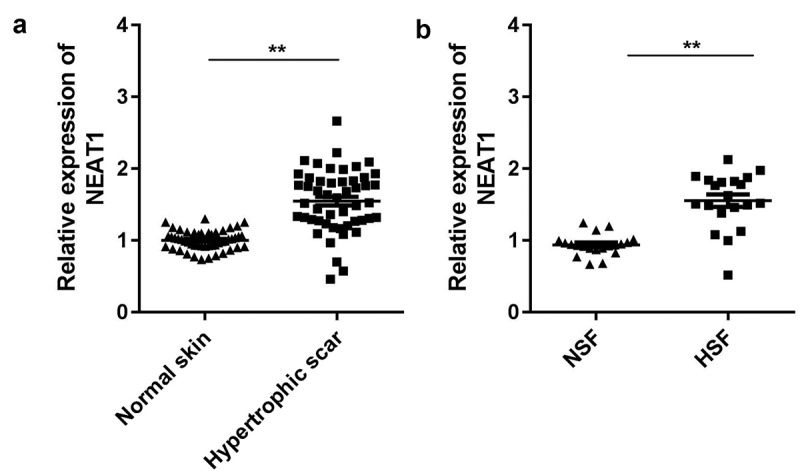
NEAT1 was upregulated in hypertrophic scar fibroblasts

As shown in Figure 1(a), NEAT1 was significantly upregulated in hypertrophic scar tissues (Figure 1(a)). This was consistent with the results of the *in vitro* assays. As shown in Figure 1(b), the expression of NEAT1 was significantly increased in HSFs. These results indicate the vital role of NEAT1 in HSF development.

A: NEAT1 expression in clinical samples was determined using qRT-PCR. B: NEAT1 expression in hypertrophic scar fibroblasts was determined by qRT-PCR. **PP < 0.01.

### NEAT1 knockdown suppressed the expression of ECM proteins

**Figure 2.**
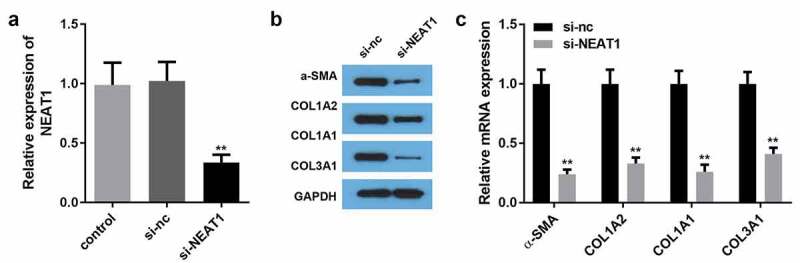
NEAT1 knockdown inhibited the production of ECM proteins

To explore NEAT1 essentiality in HSF development, we silenced NEAT1 using si-NEAT1 in HSFs. As shown in Figure 2(a), NEAT1 expression was remarkably downregulated by NEAT1 knockdown. Moreover, silencing NEAT1 drastically decreased the mRNA and protein expression of COL1A2, COL1A1, COL3A1, and α-SMA (Figure 2(b,c)).

A: Transfection efficiency was detected by qRT-PCR B: Protein expression of COL1A2, COL1A1, COL3A1, and α-SMA was determined by western blotting. mRNA expression of COL1A2, COL1A1, COL3A1, and α-SMA was determined by qRT-PCR. **P < 0.01.

### NEAT1 knockdown repressed the viability and proliferation of HSFs

**Figure 3.**
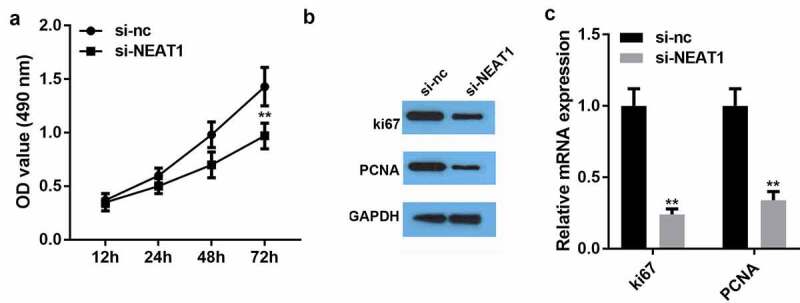
NEAT1 knockdown repressed the viability and proliferation of hypertrophic scar fibroblasts

We investigated the role of NEAT1 in the progression of HSFs. As seen in Figure 3(a), the viability assay results showed that silencing NEAT1 significantly reduced HSF viability compared to that in the negative control group. Silencing NEAT1 substantially inhibited proliferation-related ki63 and PCNA protein expression and gene expression (Figure 3(b,c)). These results indicate the crucial role of NEAT1 in HSF development.

A: Cell viability of hypertrophic scar fibroblasts was determined using the CCK-8 assay. B: Protein expression of ki67 and PCNA was determined using western blotting. C: mRNA expression of ki67 and PCNA was determined by qRT-PCR. **P < 0.01.

### NEAT1 sponged miR-29-3p

**Figure 4.**
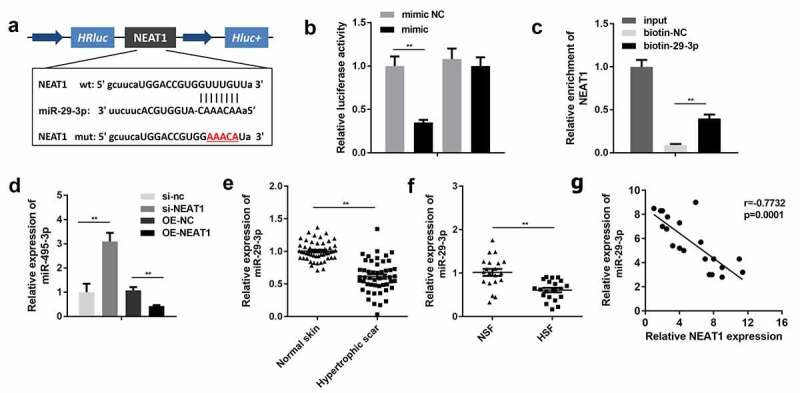
NEAT1 directly targeted miR-29-3p

Figure 4(a) shows the potential binding sites between NEAT1 and miR-29-3p. Co-transfection of wt-NEAT1 with miR-29-3p drastically suppressed the luciferase activity (Figure 4(b)). In addition, an RNA pull-down assay was performed to validate the previous results, and its results showed that NEAT1 directly bonded to miR-29-3p (Figure 4(c)). miR-29-3p was significantly downregulated by NEAT1-overexpressing plasmids and upregulated by si-NEAT1 (Figure 4(d)). Thereafter, we analyzed the expression of miR-29-3p in the normal and hypertrophic dermis. The expression of miR-29-3p was remarkably decreased in the hypertrophic dermis as well as in HSFs (Figure 4(e,f)). Moreover, NEAT1 expression was inversely correlated with miR-29-3p expression in scar tissues (Figure 4(g)).

A: Binding sites between NEAT1 and miR-29-3p. B: Interaction between NEAT1 and miR-29-3p was verified by a luciferase activity assay. C: Interaction between NEAT1 and miR-29-3p was confirmed by RNA pull-down assays. D: Expression of miR-29-3p was determined by qRT-PCR. E: Expression of miR-29-3p in clinical samples. F: Expression of miR-29-3p in hypertrophic scar fibroblasts. G: Correlation between NEAT1 and miR-29-3p expression. **P < 0.01.

### Inhibiting miR-29-3p repressed HSF proliferation and ECM production

**Figure 5.**
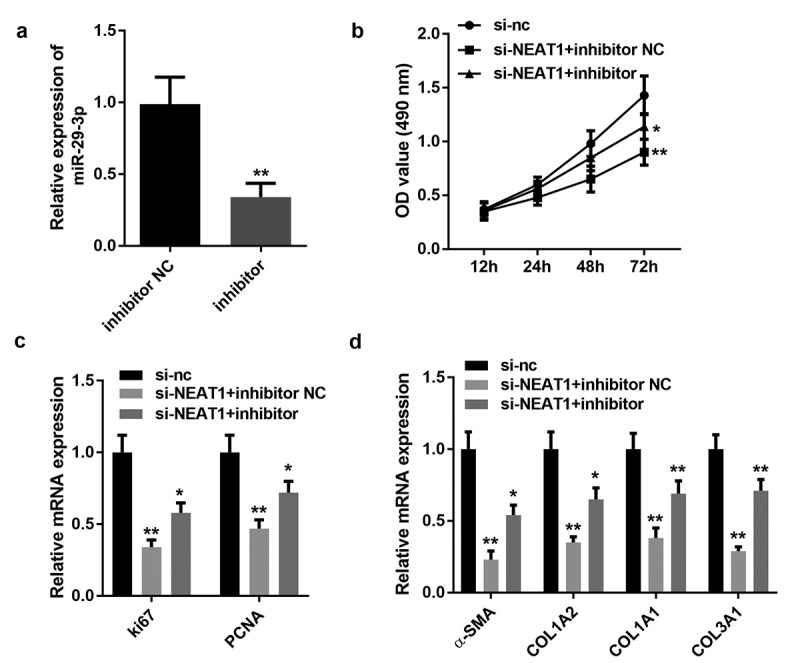
Inhibition of miR-29-3p repressed hypertrophic scar fibroblast proliferation and ECM production

Here, we investigated the biological role of miR-29-3p in HSFs. The miR-29-3p inhibitor decreased miR-29-3p expression (Figure 5(a)). As shown in Figure 5(b), the miR-29-3p inhibitor drastically inhibited HSF viability compared to that in the negative control and si-NEAT1 groups. Furthermore, treating HSFs with the miR-29-3p inhibitor combined with the silencing of NEAT1 drastically inhibited HSF proliferation, as evidenced by the inhibition of ki63, PCNA, COL1A2, COL1A1, COL3A1, and α-SMA gene expression (Figure 5(c,d)).

A: Transfection efficiency of miR-29-3p B: Cell viability was detected by the CCK-8 assay. C: mRNA expression of ki67 and PCNA was determined by qRT-PCR. D: mRNA expression of COL1A2, COL1A1, COL3A1, and α-SMA was determined by qRT-PCR. **P < 0.01.

### miR-29-3p directly targeted FRS2

**Figure 6.**
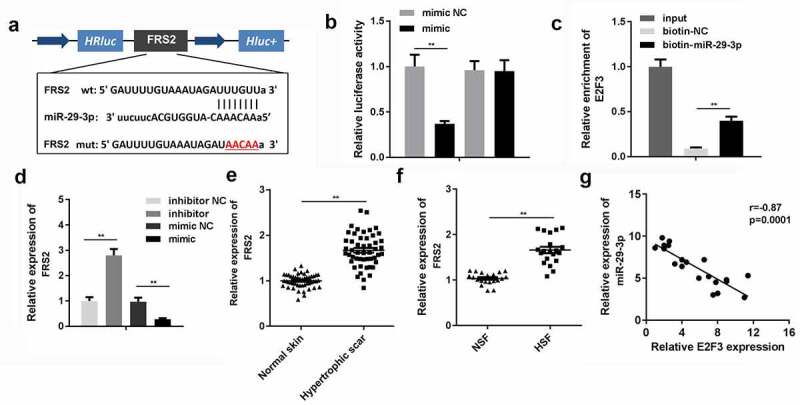
miR-29-3p directly targeted FRS2

We further investigated the underlying mechanism of the role played by NEAT1 in HSFs with respect to miR-29-3p and FRS2. Figure 6(a) shows the binding sites between miR-29-3p and FRS2. Luciferase activity and RNA pull-down assays further verified the interaction between FRS2 and miR-29-3p (Figure 6(b,c)). In addition, Spearman’s correlation coefficient analysis indicated that the expression of FRS2 was negatively correlated with that of miR-29-3p in HSFs (Figure 6(d)).

A: Binding sites of FRS2 and miR-29-3p. B: Interaction between FRS2 and miR-29-3p was verified by a luciferase activity assay. C: Interaction between FRS2 and miR-29-3p was confirmed by RNA pull-down assays. D: Expression of miR-29-3p was determined by qRT-PCR. E: Expression of FRS2 in clinical samples. F: Expression of FRS2 in hypertrophic scar fibroblasts. G: Correlation between FRS2 and miR-29-3p expression. **P < 0.01.

### FRS2 overexpression reversed the effect of miR-29-3p on the viability and proliferation of HSFs

**Figure 7.**
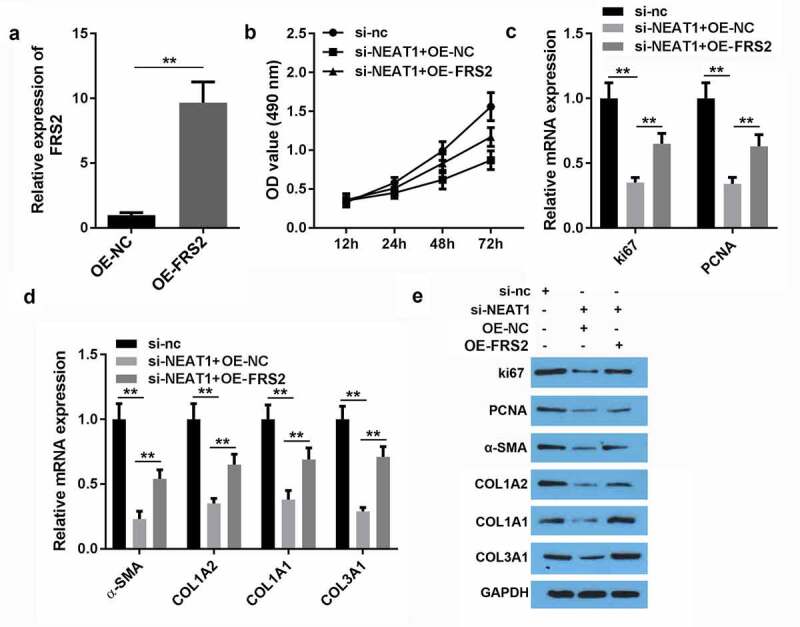
FRS2 overexpression reversed the effect of miR-29-3p on viability and proliferation of hypertrophic scar fibroblasts

We further explored the possibility that miR-29-3p may suppress HSF development by inhibiting the expression of FRS2. We transfected HSFs with either the FRS2 negative control or FRS2 vector. Transfection significantly increased FRS2 expression and significantly upregulated miR-29-3p expression (Figure 7(a)). Furthermore, overexpression of FRS2 reversed the inhibition of HSF viability induced by NEAT1 knockdown (Figure 7(b)). FRS2 overexpression remarkably reversed the NEAT1 knockdown-induced inhibition of ki63 and PCNA genes (Figure 7(c)). Moreover, silencing NEAT1 lead to inhibition of COL1A2, COL1A1, COL3A1, α-SMA, ki63, and PCNA protein expression, which was reversed on the inhibition of miR-29-3p.

A: Transfection efficiency of FRS2. B: Cell viability was detected by the CCK-8 assay. C: mRNA expression of ki67 and PCNA was determined by qRT-PCR. D: Protein expression of COL1A2, COL1A1, COL3A1, and α-SMA was determined by qRT-PCR. **P < 0.01.

## Discussion

Although various methods have been used to treat hypertrophic scarring, its recurrence has yet to be resolved. Therefore, we need to have a better understanding of the pathogenesis of hypertrophic scarring. Recently, different studies have proposed the emerging role of lncRNAs in HSF formation [7,8]. In this study, we found that NEAT1 is upregulated in the hypertrophic dermis and HSFs compared to that in normal tissues. Moreover, we identified the regulatory signaling pathway NEAT1/miR-29-3p/FRS2 in HSF development.

NEAT1 promotes distant metastasis and tumor growth in cancer [31,32]. Moreover, NEAT1 has been shown to enhance ECM accumulation and plays a crucial role in diabetic nephropathy fibrogenesis [33]. The negative regulatory role of NEAT1 in keloid formation has also been reported [14]. Therefore, NEAT1 knockdown may suppress the development of skin disorders. In this study, NEAT1 knockdown suppressed ECM release and HSF viability, indicating that the knockdown of NEAT1 may be a promising approach to inhibit HSF development.

Emerging evidence has indicated the vital role of miRNAs in HSFs, presenting them as a viable target in treatment for hypertrophic scarring [34–38]. In the present study, the lncRNA NEAT1 functioned as a competing endogenous RNA (ceRNA) to sponge miR-miR-29-3p and negatively regulate its expression. We found that miR-29-3p is downregulated in HSFs and is involved in the regulation of ECM production [20]. Moreover, miR-29-3p upregulation alleviated the effects of NEAT1 and suppressed HSF formation.

Moreover, FRS2 is required for the transduction of fibroblast growth factor receptor, which modulates various biological processes, including angiogenesis, cell proliferation, and cell metastasis [39] FRS2 has been reported to be involved in collagen synthesis [40]. Therefore, abnormal levels of FRS2 are closely associated with the development of cancer, heart disease, and neuronal disorders [21,40]. In this study, FRS2 was found to be overexpressed in HSFs. However, knockdown of FRS2 alleviated the effects of NEAT1 knockdown and suppressed the function of HSFs. To our knowledge, this is the first study to reveal the vital role of the NEAT1/miR-29-3p/FRS2 axis in HSF development, presenting this pathway as a feasible target in treatment for hypertrophic scarring.

However, there are some limitations. The role of NEAT1 in restoring epidermal integrity which is known to be important in the reduction of hypertrophic scarring in burn injury need to be further investigated. Besides, the histologic architecture of hypertrophic scars from bleomycin infusion model should be added to support the conclusion in future studies.

## Conclusion

The present study concluded that NEAT1 knockdown could repress HSF formation by inhibiting cell viability, cell proliferation, and ECM overproduction through the miR-29-3p/FRS2 axis, suggesting a feasible role of NEAT1 as an efficient target in the treatment of hypertrophic scarring.

## Highlights

1. NEAT1 was overexpressed in the hypertrophic dermis and hypertrophic scar fibroblasts (HSFs).
2. Knockdown of NEAT1 suppressed the proliferation and extracellular matrix (ECM) production of HSFs.
3. NEAT1 knockdown protected against hypertrophic scarring by modulating the miR-29-3p/FRS2 axis
